# Choriocapillaris Island: an optical coherence tomography angiography finding observed in central serous chorioretinopathy

**DOI:** 10.1186/s40942-020-00275-4

**Published:** 2021-01-07

**Authors:** Rania G. Estawro, Alaa E. Fayed, Teresa K. Gerges, Dina N. Baddar

**Affiliations:** 1 Watany Eye Hospital, Cairo, 11775 Egypt; 2 Department of Ophthalmology, Kasr Al-Ainy School of Medicine, Cairo University, Giza, Egypt; 3 Research Institute of Ophthalmology, Giza, Egypt

**Keywords:** Choriocapillaris, Central serous chorioretinopathy, Optical coherence tomography angiography

## Abstract

**Purpose:**

To report the observation of the choriocapillaris island (CCI) on optical coherence tomography angiography (OCTA) in eyes with active central serous chorioretinopathy (CSCR), and to investigate its associated clinical features.

**Design:**

Retrospective observational study.

**Methods:**

Patients diagnosed with active CSCR underwent OCTA imaging (Optovue Inc, Fremont, California, USA), and the software built-in *en face* choriocapillaris slab was examined to demonstrate CCI, defined as an area of detectable choriocapillaris flow surrounded by an area of undetectable or diminished flow. Electronic medical records (EMR) were reviewed for demographics, clinical data, other imaging modalities and any intervention, and these parameters were correlated with CCI findings.

**Results:**

25 eyes of 25 patients were recruited. CCI was detected in all examined eyes and was best elucidated on the *en face* choriocapillaris density maps. 24 eyes had focal retinal pigment epithelium (RPE) alterations overlying CCI. All 14 eyes with simultaneous fundus fluorescein angiography (FA) showed actively leaking point(s) well corresponding to the CCI location. Resolution of sub-retinal fluid in 4 eyes was associated with disappearance of CCI on follow-up OCTA scans. 1 eye showed complicating neovascularization 5 months after the initial presentation at the same location of the CCI.

**Conclusion:**

We demonstrate the observation of the “choriocapillaris island” an OCTA finding in eyes with active CSCR underneath the area of neurosensory detachment. CCI may constitute an angiographic representation of the focal area of choriocapillaris structural and functional affection, with secondary RPE alteration jeopardizing its barrier function. Larger longitudinal studies are needed to further elucidate this finding.

## Introduction

Central serous chorioretinopathy (CSCR) is an idiopathic chorioretinal disorder typically affecting young and middle-aged adults with a higher male predilection, and represents the fourth most common non-surgical retinal disease in terms of incidence [1, 2]. It was first reported by von Graefe in 1866 as a “relapsing central retinitis” characterized by serous detachment of the neuro-sensory retina at the posterior pole [3]. Since then, a plethora of clinical and histopathological studies have attempted to provide a more profound understanding of its characteristics and underlying pathophysiologic mechanisms [4, 5]. Nearly a century later, Gass was credited with characterizing its angiographic findings and coining the term “central serous chorioretinopathy” [6].

The pathophysiology of CSCR is not yet fully understood, however recent studies have suggested that leaking pachy-choroidal vessels may exert back pressure changes on the overlying choriocapillaris (CC) leading to secondary CC disruption and retinal pigment epithelium (RPE) alterations [7–10]. Localized RPE malfunction leads to focal breakdown of the outer blood retinal barrier and consequent accumulation of fluid into the sub-retinal space [2, 11, 12].

CSCR characterization has been further validated through multiple imaging modalities including fluorescein angiography (FA) [13], fundus auto fluorescence (FAF) [14, 15], optical coherence tomography (OCT) [16, 17], and indocyanine green angiography (ICGA) [18, 19]. Recently, optical coherence tomography angiography (OCTA) has been introduced as a non-invasive imaging modality enabling in-vivo angiographic visualization of different retinal and choroidal disorders [20]. This technology allows a more in-depth representation of the chorioretinal vasculature, including the choriocapillaris which plays a crucial role in CSCR pathogenesis.

The purpose of our study was to document the “choriocapillaris island”, an OCTA sign observed in the choriocapillaris slab in eyes with active CSCR, and attempt to correlate this finding with various clinical and interventional data.

## Methods

This was a retrospective analysis of patients diagnosed with active CSCR recruited in the Watany Eye Hospital (WEH) in Cairo, Egypt between January 2019 and September 2019. The study was approved by the institutional review board of Watany Research and Development Center (WRDC) and followed the tenets of the Declaration of Helsinki. A written informed consent was obtained from all participants.

### Study sample

We included eyes with active CSCR based on the clinical evaluation of a vitreoretinal consultant. The diagnosis was made by the detection of well circumscribed posterior pole neurosensory detachment on fundus examination, presence of subretinal fluid by OCT, pachy choroid or dilated choroidal vessels by enhanced depth imaging (EDI) OCT, and a leaking point (hot spot) on FA. Only eyes that had OCTA images with a quality index (Q) of 6 or more were considered eligible.

Exclusion criteria were patients with history of any systemic illnesses or medication intake that may potentially cause subretinal fluid, other vitreoretinal disorders that may confound our results, and previous ocular surgery or treatment (e.g., laser photocoagulation, intravitreal injections of anti-vascular endothelial growth factor (VEGF) or steroids). Electronic medical records (EMR ComRec Solutions Ltd. Cairo, Egypt) of recruited patients were reviewed to extract demographics and clinical information.

### Patient imaging

OCT imaging was performed using a Spectralis OCT machine (Heidelberg Engineering, Heidelberg, Germany), and the following parameters were documented for all eyes: central sub-field thickness (CST), the presence of retinal pigment epithelium (RPE) alterations, and sub-foveal choroidal thickness (SCT) using the caliber option defined as "the distance extending from the outer portion of the hyperreflective line corresponding to the retinal pigment epithelium (RPE) to the hyporeflective line corresponding to the sclerochoroidal interface" [21].

Patients who have received simultaneous fundus fluorescein angiography (FA) imaging using the Heidelberg Retinal Angiogram (HRA-2; Heidelberg Engineering GmBH, Dossenheim, Germany) were reviewed for the presence and location of actively leaking hot spot(s).

### OCT angiographic imaging and image processing

Patients underwent imaging using RTVue-XR Avanti device (Optovue Inc, Fremont, California, USA), 3D Projection artifact removal (3D-PAR) technology was used to obtain 3 × 3 and 6 × 6 mm macular scans. The *en face* image of the software built-in choriocapillaris slab (extending between the Bruch’s membrane as an upper boundary and 30 microns below the Bruch's membrane as a lower boundary) was examined by three graders (RE, TG, DB), to determine the presence of the choriocapillaris island (CCI). No subsequent image modification or processing was performed.

### CCI evaluation

**Fig. 1.**
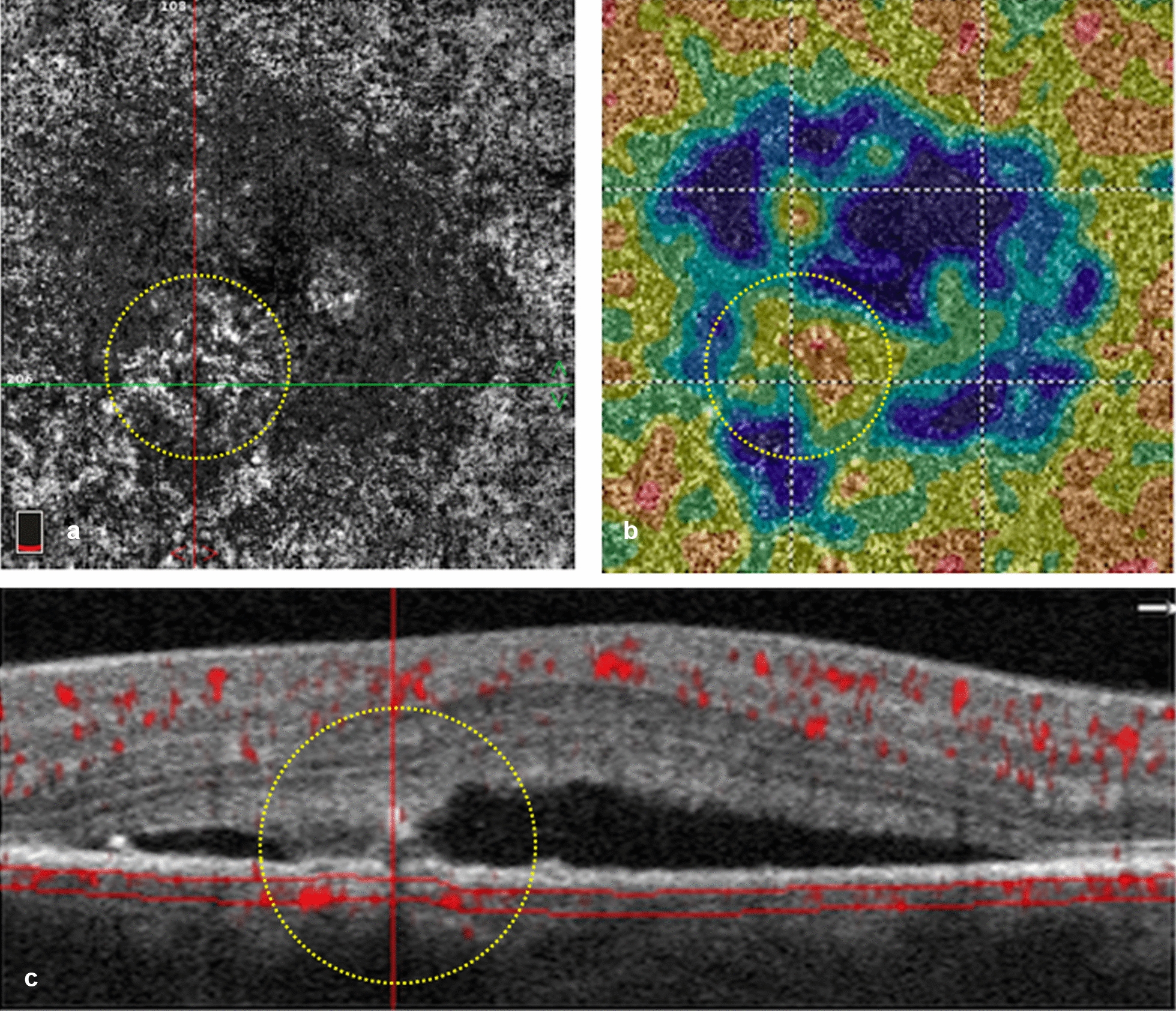
Demonstration of the choriocapillaris island(CCI) on optical coherence tomography angiography (OCTA). **a** 3 × 3 *en* face choriocapillaris OCTA showing central diminished flow underneath the area of neurosensory detachment except for a focal area (yellow circle)showing localized detectable flow, ie: the “choriocapillaris island”. **b** Choriocapillaris density map of the same area showing central signal attenuation represented in blue and an inferonasal juxta foveal CCI (yellow circle). **c** Corresponding B scan with angio-overlay showing diminished choriocapillaris flow (between red lines) underneath sub-retinal fluid except for a focal area (yellow circle) showing signal enhancement

CCI was defined as an island of detectable choriocapillaris flow surrounded by an area of undetectable or diminished flow underneath the area of neuro-sensory detachment (Fig. 1). Characterization of CCI, including the topographic location in the choriocapillaris *en face* OCTA slab and structural appearance on the corresponding cross-sectional B scan, was documented for each eye.

### Image correlation

**Fig. 2.**
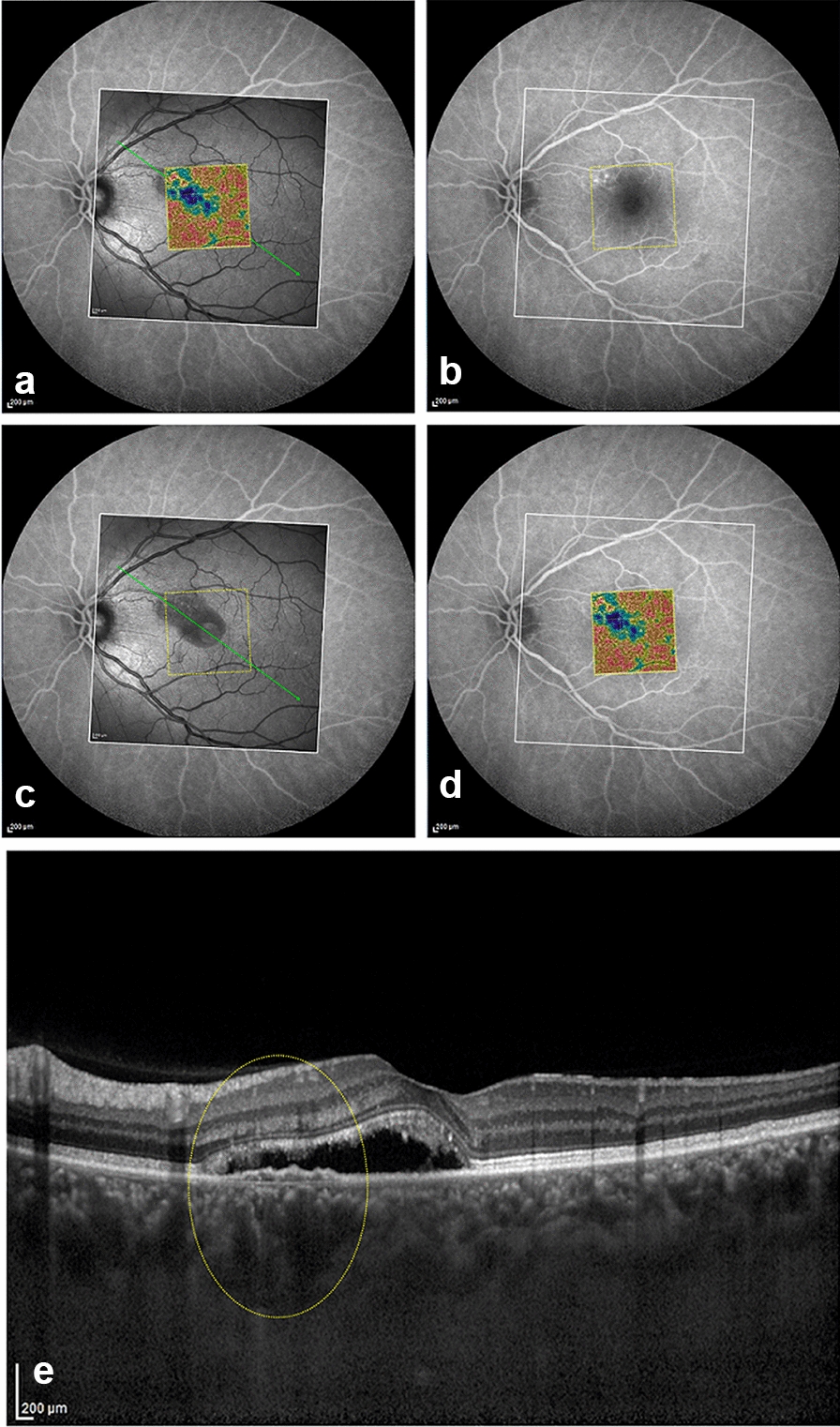
A representative example of image correlation obtained by different imaging modalities from the same patient by aligning retinal blood vessels to display the CCI location in relation to the leaking point(s)on fluorescein angiography (FA) and corresponding structural changes on optical coherence tomography angiography (OCT). **a** Superimposition of the 3 × 3 OCTA density map and the scanning laser ophthalmoscopy (SLO) OCT on FA images. **b** FA shows superonasal parafoveal leaking points, the area scanned by OCT (white rectangle) and the area scanned with OCTA (yellow rectangle). **c** Superimposition of the (SLO-OCT)image on FA image and orientation of the OCT line scan passing through leaking points (green arrow). **d** Superimposition of 3 × 3 OCTA density map image on FA image to display CCI location in relation to the leaking points. **e** Enhanced depth imaging (EDI) OCT line scan showing focal RPE alterations with underlying pachy choroidcoinciding with CCI location on density map and leaking points on FA (yellow oval)

Adobe Photoshop CS2 (Adobe Systems, San Jose, California, USA) was used to superimpose images obtained from different imaging modalities (OCTA, OCT and FA) by aligning the retinal blood vessels for each patient, to display CCI location in relation to leaking point(s) on FA, and chorioretinal structural changes on corresponding structural OCT (Fig. 2).

## Results

Out of the initially reviewed 34 eyes of 33 patients with active CSCR, 25 eyes of 25 patients (21 male and 4 female) met the inclusion criteria and were included in the study. Mean patient age was 40.6 ± 9.7 years. Mean onset of complaint was 5.2 ± 5.1 month. The Mean BCVA was 20/28 ± 20/95. The mean central subfield thickness was 417.7 ± 106.1 microns, and the sub-foveal choroidal thickness was 433.3 ± 94.6 microns. 24 eyes had retinal pigment epithelium (RPE) alterations overlying CCI.

**Fig. 3.**
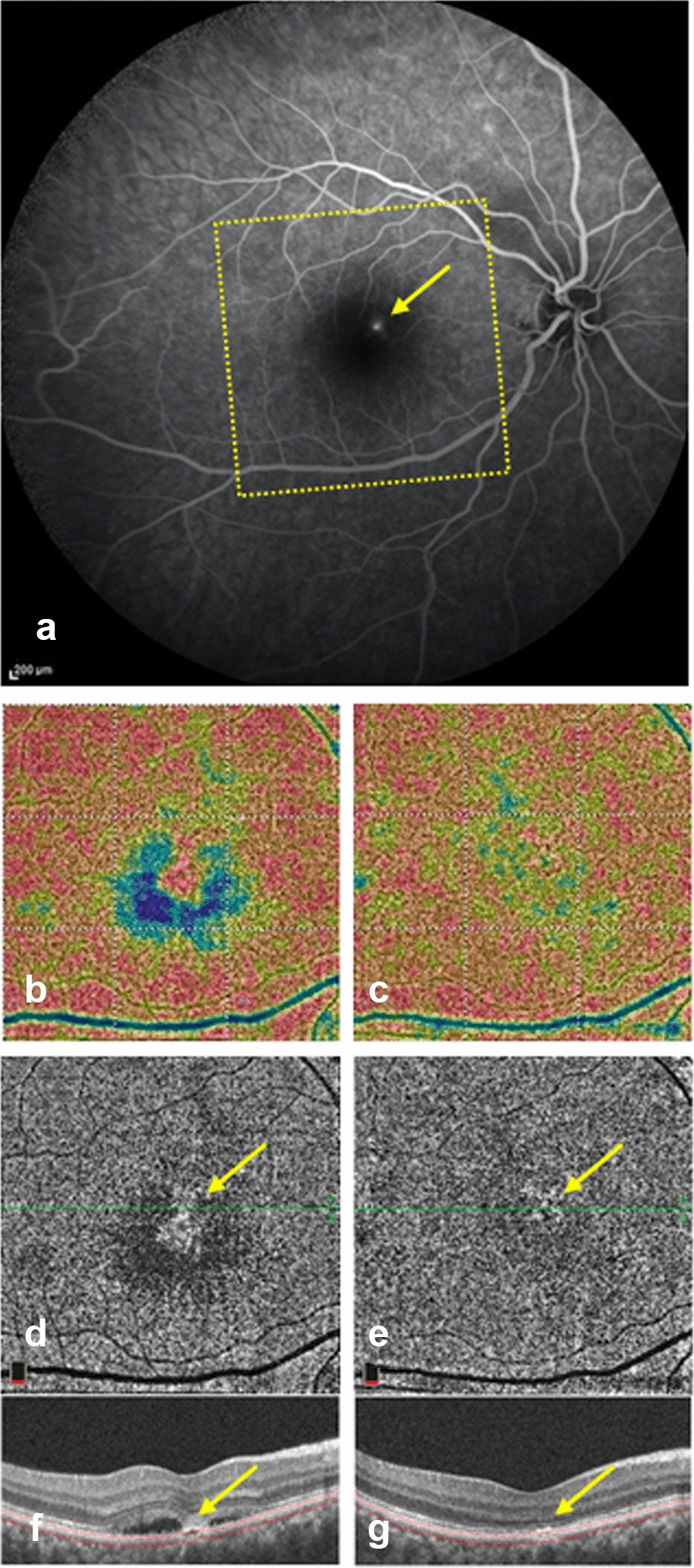
A patient showing spontaneous resolution of sub-retinal fluid within 2 months of the initial presentation. **a** FA showing superonasal a juxta-foveal actively leaking point (yellow arrow) and the area scan by OCTA (yellow rectangle). **b** 6 × 6 OCTA density map of the choriocapillaris slab at the initial presentation showing central signal void area represented in blue color and a superonasal juxta foveal choriocapillaris island (CCI) corresponding to the same location the leaking point on FA. **c** Follow-up OCTA density map showing relative restoration of the choriocapillaris flow with disappearance of CCI. **d** 6 × 6 *en face* choriocapillaris OCTA at the initial presentation showing CCI (yellow arrow). **e** Follow-up *en face* OCTA showing relative restoration of choriocapillaris flow. **f** B scan corresponding to the angiogram in D showing focal retinal pigment epithelium (RPE) changes with bridging of retinal layers(yellow arrow) coinciding with CCI. **g** Follow-up B scan corresponding to the angiogram in **e**

**Fig. 4.**
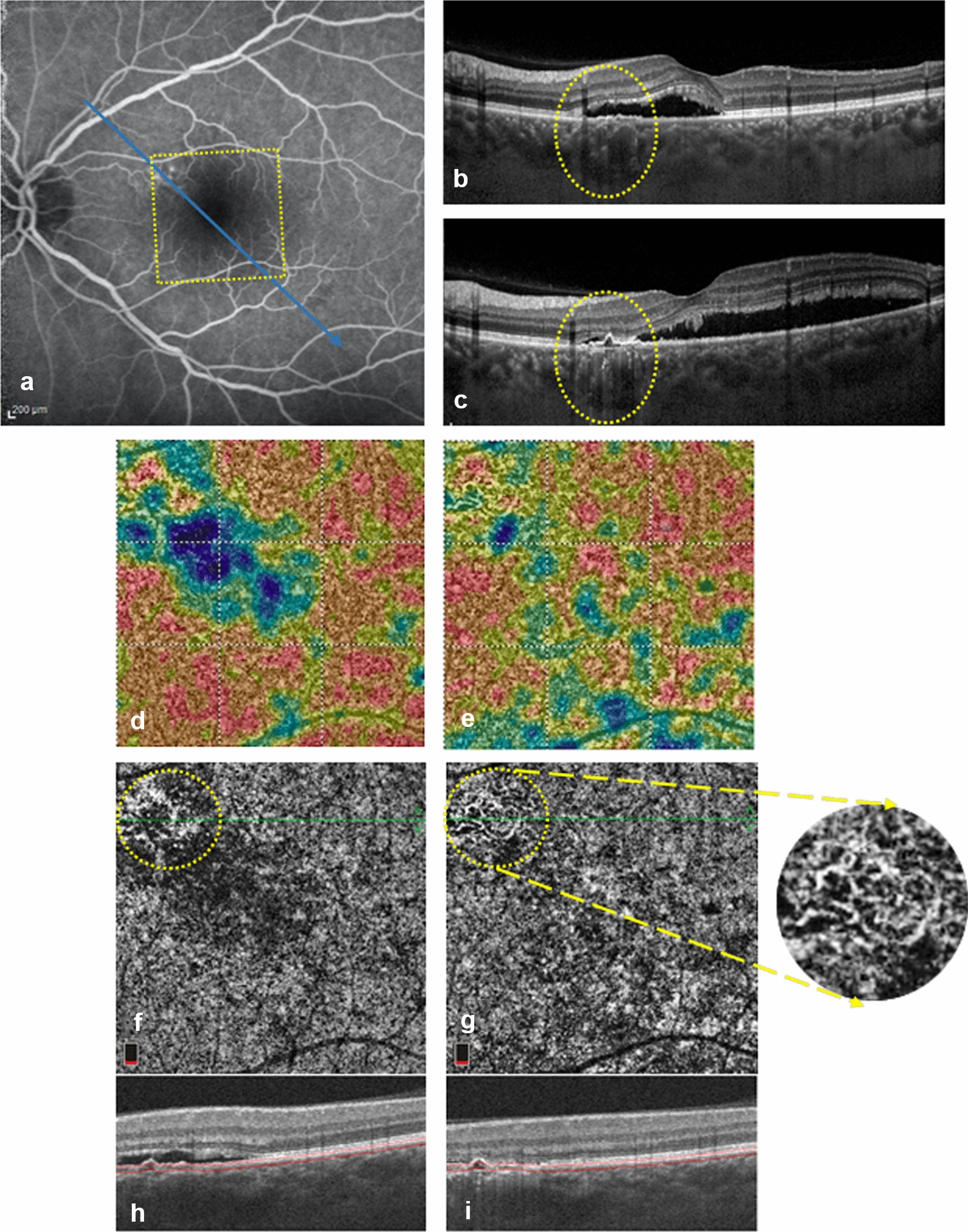
A patient showing complicating neovascularization 5 months after the initial presentation. **a** FA at the initial presentation showing superonasal para-foveal actively leaking points, the area scan by (OCTA) (yellow rectangle) and enhanced depth imaging OCT (EDI-OCT) line scan orientation (blue arrow). **b** EDI-OCT line scan at the initial presentation showing focal double layer sign and underlying pachy-choroidal vessels (yellow oval) corresponding well to th leaking points on FA. **c** Follow-up EDI-OCT line scan showing formation of a shallow pigment epithelium detachment (PED) with underlying pachy-choroidal vessels and increased sub-retinal fluid. **d** 3 × 3 OCTA density map of the choriocapillaris slab at the initial presentation showing superonasal para-foveal CCI corresponding to the same location of leaking points on FA. **e** Follow-up OCTA density map. **f** 3 × 3 *en face* choriocapillaris OCTA at the initial presentation showing CCI location (yellow circle). **g** Follow-up *en face* OCTA showing development of choroidal neovascularization (CNV) at the same location. **h** B scan corresponding to the angiogram in F showing focal retinal pigment epithelium (RPE) changes coinciding with CCI. **i**, Follow-up B scan corresponding to the angiogram in **g**

CCI was best visualized on the OCTA software-generated choriocapillaris density map, defined as the ratio of area occupied by vessels divided by the total area of the region of interest (Figs. 1b, 3b, 4d). Out of the 25 included eyes, only 14 eyes underwent FA imaging on the same visit. Comparative alignment between the 2 imaging modalities revealed that the topographic location of actively leaking hyperfluorescent hot spot(s)on FA corresponded well to the CCI on OCTA (Figs. 3, 4).

2 eyes in our study group showed spontaneous resolution of the subretinal fluid within 2 months of the initial presentation. Another 2 eyes received focal laser photocoagulation targeting the hyperfluorescent leaking point on FA with complete resolution of subretinal fluid within 2 months. Notably, evaluation of their follow-up OCTA scans revealed restoration of the choriocapillaris homogenous granular signal with disappearance of CCI on the *enface* CC slab (Fig. 3). Moreover, one of our study eyes developed complicating choroidal neovascularization (CNV) 5 months after the initial presentation. By tracking the location of the neovascular membrane, it was found to correspond to the same location of CCI detected on the baseline visit (Fig. 4).

## Discussion

In this study, we demonstrated the “choriocapillaris island” as an OCTA finding observed in the choriocapillaris slabs of eyes with active CSCR. CCI appeared on OCTA density map as an area of detectable choriocapillaris flow surrounded by an area of undetectable or diminished flow, typically underneath the area of neurosensory detachment. We were able to accurately correlate the CCI topographic location with actively leaking point(s) on FA and chorioretinal structural changes on OCT.

The choriocapillaris represents the innermost layer of the choroid underlying Bruch’s membrane. Histologically, it is composed of compacted fenestrated anastomotic capillaries, and is responsible for providing RPE and photoreceptors nourishment and removing their metabolic waste [22]. In that regard, the choriocapillaris is considered a vital component of a symbiotic unit including photoreceptors, RPE and Bruch’s membrane [23]. On the *en face* slabs of OCTA, the blood flow in the choriocapillaris layer gives off a homogenous granular appearance in normal eyes [24, 25].

In eyes with active CSCR, our data demonstrates diminished or absent CC signal underneath the area of neurosensory detachment. Although a clear explanation for this finding cannot be determined, several theories exist. One assumption is that choroidal congestion characterizing CSCR may be accompanied with sluggish choriocapillaris flow caused by mechanical back pressure changes by the dilated large choroidal vessels, thus falling below OCTA detectable threshold [26]. Another explanation is the shielding effect of the overlying detached retina and subretinal fluid leading to impairment of signal transmission, and creating false flow impairment [13, 27]. Moreover, it is possible that side walls of both the detached retina and underlying pigment epithelium detachment (PED) may provide additional sources of choriocapillaris signal attenuation. Pertaining to this theory, Zhang and associates have suggested that spectral domain (SD) OCTA may exhibit a rather limited power of signal penetration in the presence of mechanical barriers, and proposed compensated quantification of choriocapillaris flow voids using swept source (SS) OCTA in such conditions [28].

Surprisingly, inside this signal void area, we observed an island of detectable signal “choriocapillaris island” in a considerable number of eyes, which suggests a focal significantly high decorrelation value sufficient to overcome the overlying attenuating effect and can reach OCTA detectable thresholds. Interestingly, this sign can easily be overlooked on conventional *en* face CC slab, whereas it is best visualized on the OCTA density map because of the color coding which enables a more vivid appreciation of differences in flow volumes (Figs. 1b, 3b, 4d).

Several studies have demonstrated that CSCR can be classified based on the RPE changes into a classic form with focal RPE affection, and a more advanced form with diffuse retinal pigment epitheliopathy (DRPE) [29]. In 24 eyes of our study subjects, focal RPE alterations coincided with the same topographic location of CCI (Figs. 1c, 2e, 3f, 4b). Sub-foveal choroid in our study group was thickened in comparison to previously published epidemiological studies chronicling normal choroidal thickness [21, 30]. Additionally, examination of the choroid underlying CCI in the corresponding EDI OCT line scans, revealed thickening of Haller’s choroidal vessel(s) with inward displacement and focal thinning of the overlying choriocapillaris (Figs. 2e, 4b). This suggests that CCI coincides with the focal location of the chorioretinal structural affection, and further solidifies the theory connecting it to the mechanical compressive effect of the existing pachychoroid. By following up the flow void area surrounding the CCI location in larger longitudinal studies, it may be possible to demonstrate subsequent progression of RPE/choriocapillaris affection in long-standing cases [29].

Fluorescein angiography (FA) is still considered the gold standard for localization of actively leaking point(s) in CSCR [13]. In all 14 eyes of our study group who had simultaneous FA, the CCI location was closely correlated to the anatomical site of the actively leaking hot spot(s)on FA (Figs. 3, 4). These findings suggest that CCI location could be explored in larger studies as a tool to highlight areas of localized RPE functional affection, with focal breakdown of the outer blood retinal barrier, and consequent leakage of fluid from the congested choroidal vessels into the sub-retinal space. This assumption could be supported by findings reported by Maltsev and associates demonstrating that focal structural RPE alteration on OCT coincided with the leakage point on FA in a significant number of patients with CSCR [31]. In light of this observation, CCI may potentially be studied as a potential marker for guiding laser or photodynamic therapy.

Spontaneous resolution of sub-retinal fluid has been reported in eyes with CSCR eyes [2, 4, 5], while persistent cases could be management through different approaches, including focal laser photocoagulation of the leaking point [32, 33]. Two eyes of the current study group have shown spontaneous resolution of subretinal fluid (Fig. 2), while two other eyes were treated by focal argon laser targeting the leaking points on FA. All 4 eyes demonstrated complete resolution of subretinal fluid on structural OCT within 2 months of baseline examination. Notably, their follow-up OCTA scans revealed relative restoration of choriocapillaris granular appearance with disappearance of the previously detected CCI. This observation possibly reflects an enhancement of the surrounding CC signal transmission after removal of the overlying artifactual effect of the detached retina and sub-retinal fluid, and denotes restoration of the outer blood retinal barrier integrity.

Recent studies have emphasized the role of OCTA in detection of choroidal neovascularization (CNV) complicating CSCR [34, 35]. A single eye from our study group developed a secondary CNV 5 months after the initial presentation. OCTA scans confirmed the development of a branching vascular network with peripheral tiny anastomosis on the *enface* CC slab, which corresponded well to the previously observed CCI location (Fig. 4).It is possible that the longstanding anatomical and functional affection of the outer retinal layers at the CCI location, may render it most vulnerable to the development of complicating CNV. It could thus be speculated that topographic identification of CCI in CSCR patients at initial presentation, and regular follow-up using OCTA scanning of the same location, may be a useful biomarker in early detection of developing secondary CNV.

From the data provided, it cannot be ascertained whether the presence of CCI should be ascribed to focal choriocapillaris affection or to a newly forming abnormal choroidal neovascularization. However, spontaneous resolution of subretinal fluid was documented in 2 eyes, which was not previously reported in pachychoroid Neovasculopathy. When taking into consideration the unique choriocapillaris histological architecture of multiple, well separated vascular lobular units, filled and drained independently from each other, one can determine that the choriocapillaris is actually divided into multiple functional units [36, 37]. Based on this anatomical distinction, it is possible that CCI may represent a congested choriocapillaris lobule or cluster of lobules. This congestion could be attributed to increased hydrostatic pressure in pachy-choroidal vessels and sluggish drainage of affected lobule(s). In this regard, it is our opinion that CCI may possibly be an early reflection of focal choriocapillaris affection which may resolve spontaneously, or progress to complicating CNV.

The limitations of our study include its retrospective nature with lack of FA in some patients and its short-term follow-up visits, denying the opportunity to achieve more insight into the natural and treatment course of our observation in the context of evolution of CSCR. Moreover, the lack of access to ICGA did not allow a correlation of this important imaging tool in choroidal disorders with our findings. Another possible limitation is the use of SD-OCTA system that employed a shorter wavelength with a rather limited light penetration compared to SS-OCTA which utilizes a longer wave length and provides greater penetration power. However, we believe that reporting this OCTA sign would encourage further characterization and validation though larger prospective controlled studies.

## Conclusion

We demonstrated the CCI as a finding in the choriocapillaris OCTA slab in eyes with CSCR, characterized by the presence of a detectable choriocapillaris island of flow within an area of presumedly artifactual choriocapillaris flow impairment underneath serous neurosensory retinal detachment. This finding could be explored as a dye-less tool for the identification of the focal area of RPE/CC structural and functional affection in eyes with CSCR. Larger longitudinal studies are needed to provide more insight into the natural course of this finding, its prognostic implications, and its impact on various intervention modalities.

## Data Availability

The datasets used and/or analyzed during the current study are available from the corresponding author on reasonable request.

## Abbreviations

BCVA: Best Corrected Visual Acuity
CC: Choriocapillaris
CCI: Choriocapillaris Island
CNV: Choroidal Neovascularization
CSCR: Central Serous Chorioretinopathy
CST: Central Subfield Thickness
EDI: Enhanced Depth Imaging
DLS: Double Layer Sign
EMR: Electronic Medical Record
FA: Fluorescein Angiography
FAF: Fundus Auto-Florescence
ICGA: Indo-Cyanine Green Angiography
OCT: Optical Coherence Tomography
OCTA: Optical Coherence Tomography Angiography
PED: Pigment Epithelium Detachment
RPE: Retinal Pigment Epithelium
SCT: Subfoveal Choroidal Thickness
SD: Spectral Domain
SS: Swept Source
VEGF: Vascular Endothelial Growth Factor
